# Congenital Oral Squamous Cell Carcinoma in a Suckling Piglet

**DOI:** 10.1155/2021/3070559

**Published:** 2021-10-14

**Authors:** Jasmine Hattab, Abigail Rose Trachtman, Pietro Giorgio Tiscar, Marco Di Domenico, Jessica Maria Abbate, Antonio Ieni, Giuseppe Marruchella

**Affiliations:** ^1^University of Teramo, Faculty of Veterinary Medicine, Loc. Piano d'Accio, 64100 Teramo, Italy; ^2^Istituto Zooprofilattico Sperimentale dell'Abruzzo e Molise “Giuseppe Caporale”, Campo Boario, 64100 Teramo, Italy; ^3^Department of Veterinary Science, University of Messina, Polo Universitario dell'Annunziata, 98168 Messina, Italy; ^4^Department of Human Pathology of Adult and Evolutive Age “Gaetano Barresi”, Section of Pathology, University of Messina, 98125 Messina, Italy

## Abstract

A 3-week-old suckling piglet spontaneously died after septicemic colibacillosis. At postmortem examination, bulging and ulcerated lesions were seen, affecting the oral mucosa on the inner surface of the lower lip. After histopathological investigation, the diagnosis of congenital oral squamous cell carcinoma was made. To the best of our knowledge, this is the first case of congenital oral squamous cell carcinoma ever described. A relationship has been shown or suggested between papillomavirus infection and oral squamous cell carcinoma in humans and animals. However, next-generation sequencing study did not demonstrate any papillomavirus sequences in the case reported herein.

## 1. Introduction

Cancer is widely recognized as a major cause of morbidity and mortality in small companion animals [1]. The occurrence of cancer is much less frequent in farm animals, a few exceptions being represented by tumors caused by oncogenic viruses (e.g., ovine pulmonary adenocarcinoma sustained by Jaagsiekte sheep retrovirus) or by environmental and/or genetic factors (e.g., ocular squamous cell carcinoma in white-faced cattle chronically exposed to sunlight) [2, 3]. Overall, cancer is rarely detected in commercially reared pigs, with a prevalence of less than 40/10^6^ at slaughter, likely due to their short lifespan. Lymphosarcoma, nephroblastoma, melanoma, and hepatic tumors are considered to be the most common porcine neoplasms, while other tumors are only sporadically observed in this animal species [4]. The relevance of age as a risk factor is strengthened by the much higher frequency of tumors observed in pet pot-bellied pigs [5]. The present report is aimed at describing the main pathological features of a case of oral cancer in a suckling piglet.

## 2. Case Presentation

The event occurred in a small farrow-to-finish pig herd located in central Italy, the breeding stock consisting of 70 sows (Landrace x Large White x Duroc) and 2 boars (Mora Romagnola, an Italian local breed). All pigs were routinely vaccinated against Aujeszky's disease (Aujeszky A-Suivax GI, Fatro, Italy), according to the Italian law. In addition, sows were vaccinated against colibacillosis and clostridiosis (Suiseng, Hipra, Spain), while piglets were vaccinated 15 days from birth against porcine circovirus type 2, *Mycoplasma hyopneumoniae*, and porcine reproductive and respiratory syndrome virus (3FLEX®, Boehringer Ingelheim, Germany).

In May 2020, the herd experienced a severe episode of colibacillosis in suckling piglets, with a relevant increase of mortality. Several piglets were autopsied for diagnostic purposes. Among them, a 3-week-old piglet was examined. The animal showed a moderate body condition. At necropsy, acute enteritis, systemic enlargement and hyperaemia of lymph nodes, mild splenomegaly, pulmonary oedema, and disseminated petechiae on the visceral pleura, as well as on the external surface of the kidneys, were seen. On the basis of the postmortem pathological findings, septicemia was suspected. Bacteriological investigation yielded the isolation of *Escherichia coli* in pure culture from the spleen, thus supporting the diagnosis of septicemic colibacillosis.

**Figure 1:**
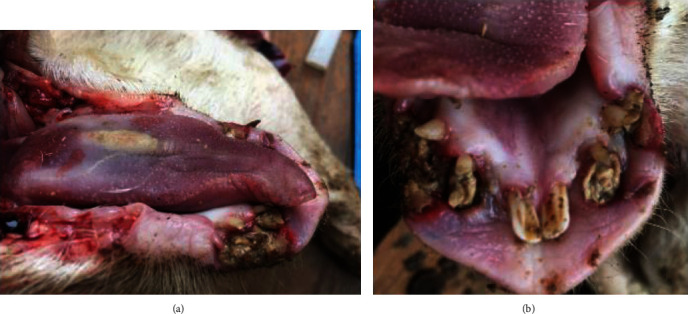
Suckling piglet. Gross pathology. A large lesion (2 × 1.5 cm) was seen on the inner surface of the lower lip, close to the 2^nd^ and 3^rd^ right incisors. The lesion was ulcerated and protruded onto the surface of the oral mucosa (a). Ulcerated and smaller lesions were additionally detected close to the 2^nd^ and 3^rd^ left incisors (b).

**Figure 2:**
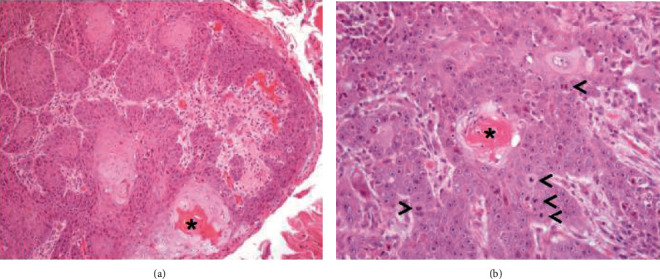
Suckling piglet. Oral lesion. Histopathology. Cords and islands of neoplastic cells almost entirely infiltrate the lamina propria of the oral mucosa (a). Inside the proliferation, neoplastic cells undergo keratinization and tend to arrange as concentric layers, thus forming a horn pearl draft (black asterisk). At this magnification, prominent nucleoli are also evident, as well as several mitotic figures (black arrowheads; (b)). H&E, original objective 10x (a) and 20x (b).

**Figure 3:**
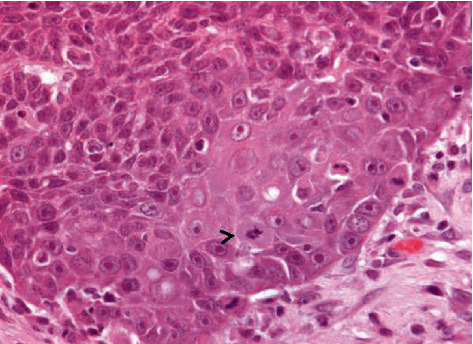
Suckling piglet. Oral lesion. Histopathology. At higher magnification, intercellular bridges can be easily appreciated (black arrowhead). Mitoses are also evident. H&E, original objective 40x.

**Figure 4:**
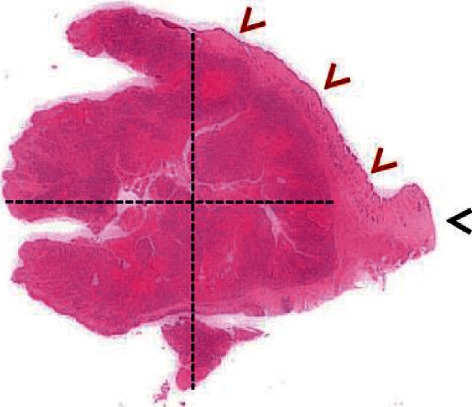
Oral lesion. Scanning of the entire slide. This picture clearly shows that the neoplasm was very extensive in both width and depth (black dotted lines). The lesion widely infiltrated the lip thickness, excluding only a thin layer of skin (red head arrows) and the lip border (black head arrow).

Notably, bulging and ulcerated lesions were also seen, affecting the oral mucosa. The largest lesion was seen on the inner surface of the lower lip, close to the right second and third incisors (Figure 1(a)), while smaller and mainly ulcerative lesions were observed in correspondence with the second and third incisors on the left side (Figure 1(b)). Considering their appearance and location, such lesions were thought to have resulted from traumatic injuries complicated by bacterial infection. Representative tissue samples were collected from the oral lesions, promptly fixed in 10% neutral buffered formalin, and routinely processed for histopathological investigation (hematoxylin and eosin stain, H&E). The microscopic examination of oral lesions conclusively demonstrated the presence of a nonencapsulated neoplastic proliferation, consisting of large, polygonal epithelial cells, provided with a single nucleus with a prominent nucleolus and abundant cytoplasm. Neoplastic cells were arranged as cords and islands, leaking from the epithelium and infiltrating the underlying lamina propria (Figure 2(a)). Keratinization of individual epithelial cells and so-called “keratin pearls” were detected throughout the tumor. A very high number of mitoses (33 × 10 contiguous fields, high-power field 40x original objective), sometimes atypical, were observed (Figure 2(b) and 3). In addition, intercellular bridges were clearly seen between adjacent neoplastic cells (Figure 3). Inflammatory infiltrates, mainly consisting of lymphocytes and occasionally arranged as lymphoid follicles, were present at the periphery of the neoplastic proliferation. As shown in Figure 4, the neoplasm invaded almost the entire thickness of the lip, while it did not affect the underlying bone tissue.

On the basis of the above microscopic findings, taking into account that tumors are defined as congenital when detected in foetuses and newborns until 2 months of age [4], the final diagnosis of congenital oral squamous cell carcinoma (SCC) was made. Farrowing room staff did not notice such oral lesions at birth or in the following days, during routine handling (e.g., iron injections). However, we consider unlikely the development of such large neoplasms in a few weeks, after birth.

Unfortunately, we did not sample any other tissues or organs; thus, we cannot rule out the presence of metastases. We nonetheless remark that no microscopic evidence existed regarding the presence of neoplastic emboli inside the lymphatic and blood vessels; furthermore, lesions resembling neoplasms were not grossly observed at postmortem examination.

Thereafter, an aliquot of the formalin-fixed tissue sample was submitted for an in-depth biomolecular investigation, in order to confirm/rule out the presence of papillomaviruses or, more in general, oncogenic viruses. To this aim, DNA was extracted by the Maxwell® RSC DNA FFPE Kit (Promega, Madison, WI), using the Maxwell 16 Instrument (Promega, Madison, WI), according to the manufacturer's protocol. Genomic libraries were prepared by means of the Illumina DNA Prep (Illumina, San Diego, CA) and then sequenced on the MiniSeq Instrument (Illumina Inc.), using MiniSeq Mid Output Reagent Cartridge v2 (Illumina Inc.), 300 cycles, and standard 150 bp paired-end reads. Raw reads were trimmed by Trimmomatic [6], and shotgun metagenomic analysis was accomplished by Kraken [7]. Metagenomic analysis at genus rank revealed 35 common contaminant bacteria and 12 viruses. The most abundant ones were *Bacillus* spp. (1718 reads) and circoviruses (360 reads), respectively, followed by less represented taxa. No papillomaviral DNA sequences were found in any of the reads.

## 3. Discussion

Oral SCCs are commonly diagnosed in cats and dogs, much less frequently in horses and sporadically in sheep [8]. In general, oral SCCs are also considered rare in cattle, with the exception of some geographic areas (e.g., Southern Italy) where they can result from a simultaneous infection with bovine papillomavirus type 4 and exposure to bracken fern (*Pteridium aquilinum*) [9]. In pigs, erosive or ulcerative stomatitis can result from viral vesicular diseases [10]; in this respect, we remark that Italy is currently free from such disease conditions (data available at the website of the OIE World Organisation for Animal Health, http://www.oie.int). More commonly, cases of posttraumatic, necrotizing stomatitis by *Fusobacterium necrophorum* are observed in piglets. Formerly, traumatic injuries often resulted from tooth clipping; such a procedure was widely abandoned in Italy and not performed in the pig herd studied. As above stated, we also mistakenly suspected posttraumatic lesions. However, microscopic findings leave no margin for doubt, as further confirmed by two skilled veterinary pathologists, who remained blind to signalment and case history.

Two cases of oral SCC have been described thus far in pigs, both affecting aged pet pot-bellied pigs. The first case was observed in a 10-year-old female pig, and SCC appeared as an ulcerated mass which infiltrated the hard and soft palate and metastasized to the draining lymph node and to the lungs [11]. The second case was detected in an 18-year-old, neutered male, Vietnamese pig and was located lateral to the right mandible; inside the oral cavity, no evidence of metastasis was being noted [12]. Therefore, the present report describes an exceptionally rare disease condition and, to the best of our knowledge, it represents the first case of a congenital oral SCC ever described.

As reviewed in detail in a previous work, porcine congenital tumors are uncommon and mostly of mesenchymal origin. Despite their very low prevalence, some congenital tumors in pigs (i.e., melanomas and lymphomas) have been studied thoroughly as models in comparative oncology, because of their mode of inheritance [4].

On an interesting note, a report regarding newborn piglets in China showed a very high incidence of enzootic lingual papillomatosis (>100.000/year) [13]. We consider this to be very intriguing seeing as how (a) papillomaviruses are able to cross the placental barrier [14, 15]; (b) congenital papillomatosis have been reported in different animal species [16–20]; and (c) a relationship has been shown or suggested between papillomavirus infection and oral SCC in humans [21] and animals [9, 22]. Knowledge about suid papillomaviruses (SPVs) is scant, a single case of cutaneous fibropapilloma having been associated with SPV infection in a wild boar [23]. Laboratory data herein provided does not support the etiologic involvement of SPV, as well as of other oncogenic viruses in the present case report. More sensitive tests (e.g., real-time polymerase chain reaction) could be useful to better detail the role, if any, of SPV in the pathogenesis of similar neoplasms. However, we remark that no further warts and/or neoplastic lesions have ever been detected in the farm under study.

Overall, infectious diseases represent the most relevant issue in the field of porcine health management, leading the diagnostic approach to mostly be focused on viral and bacterial pathogens, often by means of biomolecular tools. Histopathology is not routinely carried out for diagnostic purposes in farm animals, at least in the Italian context. In spite of this, the present case report highlights that microscopic investigation is always of value to integrate with other laboratory tests, allowing for a more correct understanding of misleading postmortem findings and providing more reliable data about the prevalence and the main features of cancer in the different animal species.

## Data Availability

All data are included in the main text of the manuscript.
